# Evaluating Instructor Feedback Practices in a Clinical Learning Environment in a Developing World Setting; a Qualitative Study

**DOI:** 10.21203/rs.3.rs-5415132/v1

**Published:** 2024-12-04

**Authors:** Moreen Tumwine, Boaz Mucunguzi, Guti Walker, Ian Munabi, Aloysious Mubuuke, Sarah Kiguli

**Affiliations:** Kampala International University; Makerere University; Makerere University; Makerere University; Makerere University; Makerere University

**Keywords:** Feedback, workplace-based learning, clinical teachers, clinical instructors, clinical rotations, clinical tutors

## Abstract

**Background:**

Feedback is an essential component in medical education. Delivering timely and relevant feedback to individual students enhances workplace-based learning. There is increasing evidence that current feedback practices are not fit for purpose. However, there’s paucity of data on studies evaluating how feedback is given by tutors to students in developing countries. The study sought to evaluate the feedback practices of tutors of clinical medicine students in the facilitation of workplace learning.

**Methodology:**

It was a qualitative study design. The study was conducted at Kampala International University in Tanzania, KIUT. The study participants were clinical medicine (14) tutors and (22) third year technician certificate in clinical medicine students. Data was collected through In-depth Interviews. The transcribed data was analyzed by inductive thematic analysis

**Results:**

This study revealed five key themes: Feedback Delivery and Approach, Feedback Timing, Feedback Content, student Engagement and Participation, Challenges in giving feedback. Most tutors provided constructive, timely, and engaging feedback. The challenges dominantly were; Students not being receptive to feedback, Individual differences. Diverse learning styles, student resistance to act on feedback. Notably, most of the students were dissatisfied with the feedback provided citing its absence, delay, inconsistency, discouraging comments, and harsh language used by certain teachers.

**Conclusion:**

Generally, the clinical tutors provided feedback that was constructive and student engaging but lacked the crucial aspect of observed performance and student reflection; hence an incomplete feedback process. Moreover, a majority of students expressed dissatisfaction with it. To improve impact of feedback on student learning and growth in clinical settings, institutions should implement Faculty Development Programmes that will provide training and support to tutors on effective feedback practices.

## Background

Workplace-based learning, rooted in the apprenticeship model, is considerably the most effective intervention of translating medical theory into clinical practice [1]. Modern clinical training coupled with competency based education and programmatic assessment methods, is focused on assessment and feedback on routine tasks in the workplace; aiming at the highest level of Miller’s pyramid [2, 3]. Hospitals, clinics and communities provide ideal environments for students to apply the classroom obtained medical knowledge to practice and develop competence. The vital component of good workplace learning is supported participation as guided by the ‘FAIR’ model. This model is guided by four principles: that is learners benefit most when provided appropriate ‘Feedback’ while ‘Actively’ participating in clinical activities with attention to ‘Individualization’ and ‘Relevance to the respective student needs[1].

Feedback is an essential component in medical education as it enhances student development and learning by providing valuable information which in turn improves their performance. There are different types of feedback as demonstrated in various teaching scenarios whereby it can either be peer assessment or from instructors[4]. Feedback can come in many forms such as praise, cues, goal setting and corrective using different mechanisms such as computer based, written or face to face[5]. Well as feedback can be formative or summative, this study focused on formative instructor feedback that is given frequently during a learning process to facilitate and improve the learning experience. Furthermore, active student reflection and constructive feedback from clinical instructors is a pillar of effective workplace learning. This is critical to the development of student competence and confidence[6].

One of the mostly used feedback model is Pendelton feedback- that developed by Pendleton as described in his book “The Consultation” this style is supported by the learner-centered models of feedback that place the learner at the center of the feedback conversation. It’s of a high educational value, straightforward, dialogues based and easy to reproduce. It starts with student reflecting on their performance, trainer giving feedback, a discussion for improvement and concludes with making an action plan/strategy for improvement[7].

Despite the role of feedback in the teacher- learner interaction, institutions of higher learning continue to face criticism for the inadequacies in the feedback they provide to students[8],[9]. Reportedly [10] some clinicians gave feedback in a very disrespectful manner which was discouraging and humiliating in front of a patient. It has been severally reported that health professional students are rarely directly observed and given feedback during their clinical placements[3]. In a study of [8], it was noted that feedback practices are often unsustainable which is demotivating for students.

At KIUT, it’s not known whether and how the teachers provide feedback to their students during the workplace-based learning. Therefore, this study sought to evaluate the existing feedback practices as employed by the clinical teachers/instructors in the facilitation of workplace learning of clinical medicine students at KIUT and highlight theoretical principles applicable to feedback practices.

## Methodology

### Study design

This was an explorative qualitative study design.

### Study setting

The study was conducted at Kampala international university in Tanzania (KIUT), a private university in Dar es Salaam, Tanzania. It has various faculties including faculty of Allied Health Sciences which houses the clinical medicine program in the department of clinical medicine. Clinical medicine, CMT is a 3 years diploma program that runs a competence-based vertically integrated curriculum. The clinical medicine students are introduced to clinical rotations as early as first year in the first semester until the final year of their studies. During each semester students undergo theoretical and clinical training-workplace based learning at various hospitals assigned by the university. The teaching hospitals include; Temeke Regional Referral Hospital for 3rd year students, Kisarawe, Chanika District Hospitals and Pugu Health Centre for 1st and 2nd years. During clinical rotations, the clinical teachers/instructors are supposed to closely supervise the students as guided by the curriculum so as to attain the target competencies.

### Study participants

The target population was clinical medicine tutors at KIUT involved in the supervision of students in the clinical rotations/placements as well as third year clinical medicine. The study participants (tutors) were recruited by convenient sampling method because during the study period, the tutors had been assigned to supervise students during their clinical rotations at various nearby hospitals. Therefore, the researchers considered those tutors who were available and accessible for participation in the study. The 14 and 22 participant sample size of tutors and students respectively was determined by the point of saturation.

### Data collection

Data was collected using in-depth interviews. Each IDI was conducted individually for a duration range of twenty to thirty minutes. Data was collected from 24th of April to 7th June 2023 by a two-member research team that consisted of the principal investigator(PI) and a research assistant (RA). The PI arranged appointments with the respective participants via phone calls for tutors and Class representatives for the students. Once the participants consented to the study, the RA conducted the interviews using an Android mobile phone aided with an app called TransVoice.

### Data analysis

The data analysis process involved a step-by-step approach, where each question’s responses were analyzed separately by the research team (PI and data analyst). Codes were then assigned to each participant’s response, and subsequently, these codes were condensed to identify and generate themes.

## Results

The aim of the study was to evaluate the feedback practices of tutors of clinical medicine students in the facilitation of workplace learning.

### Participant Demographic characteristics

A total of 14 tutors participated in the study, with a representation of eight 08 males and six 06 females. Whose teaching experience ranged from 1–16 years with the majority having less than 4 years of teaching experience. Most of the tutors were below 30 years of age. The students in total were 22 with 12 males and 10 females from the 3rd year clinical medicine with a median age of 23 years; hence a total of 36 participants for the entire study.

### Theme 1: Feedback delivery and approach

Instructive feedback and observation-based assessment, constructive feedback, recognition and encouragement were expressed as the approaches the clinical teachers were using in delivering feedback.

Initially, I train them on how to perform procedures. Then, I assign them tasks and observe their performance to gather feedback. This helps me understand their progress and how well they continue their studies… The feedback I’m referring to is what we call instructive feedback. For example, when a student presents their work, I provide feedback on their performance. (SNt.01).

The study findings indicated that most tutors were assigning patients/cases to students for clerkships after which they would present and be given feedback on how they performed.

Usually, I give feedback to students when they present a case and during discussions on different parts of the history and examination. I provide positive feedback as well as constructive criticism to help them identify areas for improvement. Sometimes, I may suggest they observe a new physical examination and provide feedback after they have performed it.(SNt.02)

Constructive feedback: students appreciated the aspects of feedback that focused on identifying areas of improvement and offering guidance as expressed below.

Based on my experience with feedback from my teacher, I found that it greatly contributed to my improvement. For instance, during the clinical skills training, my teacher instructed us to interact with patients, gather their case information, and present it to her for evaluation. Through this process, she identified and corrected our mistakes. Initially, I lacked confidence in communicating with patients and making fewer errors. However, with each subsequent interaction, I noticed a significant improvement in my skills (SNs.08)

#### Theme 2-Feedback timing.

Participants stated that they were giving immediate feedback and ensuring collaborative learning in group settings.

Okay, the most common way is to provide feedback. I give feedback to students, especially during their presentations. After the presentation, I give the student feedback, explaining both the areas they have done well and the areas that need improvement. This helps them perform better the next day, whether it’s an assignment or another activity (SNt.06)

Immediate feedback: some students expressed their satisfaction with the immediate feedback they had received from their clinical teachers.

During clinical examinations, the feedback is immediate, as the tutors provide corrections and suggestions on the spot (SNs.09)

Delayed or inconsistent feedback: some students expressed frustration with this aspect of the feedback they received during rotations as being delayed and inconsistent which didn’t match with what usually happens during their long case clinical examinations.

Yes, some clinical instructors provide us with feedback, but not all of them. After our rotation sessions, they give us some feedback, but it’s not consistent across all instructors. As students, we may not have extensive knowledge about patient care, so when some teachers do not give us feedback, as a result, we end up not learning how to perform certain procedures or examinations (SNs.18)

Personally, I haven’t received much feedback. However, the feedback I did receive was valuable as it pointed out my mistakes, allowing me to learn from themSNs.12

### Theme 3: Feedback content

Tutors indicated how they used feedback to evaluate student comprehension and providing tailored feedback to address individual needs by actively listening, comprehensively assessing, and identifying gaps in knowledge to provide targeted feedback.

Well, in clinical rotations, we have different settings for learning. Firstly, I have to understand the students and assess if they truly understood what they were told. After that, I give them a chance to ask questions. From there, I know where to start and what they need to know (SNt.08)

Sure. For example, when a student presents a case or performs an examination, I evaluate their physical examination skills. If the student performs well, I provide positive feedback. However, if they make mistakes, I address those areas and suggest improvements. I guide them on what not to do and what they should do instead. This approach helps them learn (SNt.12)

Discouraging feedback: some students felt the feedback was rather discouraging than encouraging as expressed below;

One instance stands out during a history taking session; the feedback I received was predominantly negative, especially regarding the general examination. It was disheartening because I felt that I had performed well in other areas of the clinical exam. The feedback I received indicated that I needed improvement in the HPI and general examination, and that was the only aspect mentioned (SNs.16)

Moreover, others felt the language used was harsh:

We have been receiving some feedback from our teachers. Initially, there was some feedback that was a bit harsh for us. Whenever we made a mistake, the teacher would criticize us openly, creating an uncomfortable situation, especially in front of patients. This approach not only affected our confidence but also undermined the trust of the patients. I don’t think that’s appropriate (SNs.17).

### Theme 4: Engagement and participation

Participants said they were involving students in the feedback process, promoting collaboration and active participation.

…. each student takes turns to present, and the rest listen. If anything, essential is missing for them, I make sure to address it during my teaching session (SNt.04).

After the presentation, as a teacher, I listen to the students and their peers to understand their perspectives. Based on their responses, I provide feedback on their approach to clerking the patient, both on what was done correctly and what was not supposed to be asked or documented (SNt.05).

Students pointed out limited opportunities of participation due to multiple colleges at the clinical site;

It’s more about the number of students and the limited opportunities to interact with patients during rotations. For example, when we go to hospitals, there are many students, and it becomes difficult for the doctor to attend to everyone. Only a few students get the chance to showcase their skills (SNs.16).

### Theme 5-Challenges

The study defined the “challenges faced in the feedback delivery” as the shortcomings the teachers were facing in delivering feedback and possible hinderances to feedback serving its effectual purpose.

Some respondents expressed concerns about students not being receptive to feedback or not diligently completing assignments and perception of feedback as criticism.

There are certain challenges that arise while providing feedback. One common challenge is when students repeat the same mistakes despite receiving feedback. For instance, if multiple students are making the same error during a particular exercise, it can be frustrating as an educator (SNt.05)

One challenge is that some students may not receive feedback positively. They might interpret it as criticism rather than help (SNt.03)

Respondents recognized that each student had their own unique way of understanding and processing information which posed a challenge in delivering individualized feedback tailored to specific needs.

One challenge I often encounter is addressing individual differences and varying learning styles. Each student has their own unique way of understanding and processing information, so tailoring feedback to meet their specific needs can be challenging. Additionally, managing time constraints and providing timely feedback to a large number of students can also pose difficulties (SNt.09)

Some tutors expressed concerns about student commitment, punctuality, and preparedness in clinical rotations. Citing that some students were not consistent in attendances which lead to unnecessary repetition of similar concepts.

One challenge is that some students simply ignore or do not take notes when I provide feedback. They may not complete the assigned tasks or fail to act upon the feedback given. Consequently, we end up repeating the same discussions and starting from scratch (SNt.11)

Moreover, tutors expressed issues such as big numbers, hesitancy in expressing feedback needs, time constraints, large group feedback.

Additionally, there are situations where we have a large number of students, making it impossible to give individual feedback. Instead, we resort to giving feedback to groups of students who might have similar challenges (SNt.07)

Some tutors felt they needed support or strengthening on how to provide feedback by citing how they had been sub consciously doing it but not quite certain whether it was being done right.

Regarding feedback in a clinical rotation, I think we have been practicing it without realizing its importance. Perhaps there should be more discussion and emphasis on its effectiveness. We should strive to improve our feedback practices(SNt.08)

However, students expressed the challenges of tutor absenteeism and inconsistencies in clinical rotations hindrance to effective feedback;

Well, on my side, I can say that the feedback is very poor, because the clinical instructors from our university, when they most of the time come to the clinical sites, they just come to sign the log books that we are signing for clinical rotations. They just leave us most of the time, they don’t come and see what we are doing inside the clinical sites, they just come to supervise the attendance of the people who have attended on the clinical rotations (SNs.05)

Non-progression; some students felt the feedback they were receiving from their teachers was not impactful as they were not observing an improvement in their performances.

…. I haven’t seen significant improvement despite receiving feedback. I feel stuck at around a 10–20% improvement rate. I continue to make the same mistakes and haven’t found effective solutions (SNs.15).

## Discussion

The study sought to evaluate the feedback practices of clinical teachers(tutors) of clinical medicine students in the facilitation of workplace learning at KIUT.

Most of the tutors were giving constructive, timely and student engaging feedback which aligns with some characteristics of effective feedback practices as indicated in literature [11, 12].This good practice could be attributed to either student feedback to their respective teachers, passion for teaching that motivates a teacher to always seek better ways of instructing and engaging students or the teacher was well versed with what the curriculum required. This study however cannot relate it to training as this aspect was not evaluated.

Well as somewhat characteristically effective feedback was being provided, it was noted that most of the tutors did not directly observe their students’ performances when providing feedback. Instead, they mentioned that they would listen to students’ case presentations and provide feedback. However, this approach does not involve observing how a student actively interacts with a patient during clerkship, for example. This lack of direct observation may have implications for accurately assessing students’ abilities and providing targeted feedback. This finding corelates with studies [3, 13] which reported that students are rarely observed.

From the findings, less than half of the students were satisfied with the feedback they received and these appreciated the Personalized and specific, Immediate, Constructive nature of feedback provided. The findings emphasize the significance of providing constructive feedback to maximize the impact on learning. Constructive feedback creates a supportive and encouraging environment that fosters students’ receptiveness to feedback and motivation to work on their shortcomings.

Upon analysis of the tutors’ feedback practices; none of them involved “student reflection on their performance ”,a key component in the feedback process embedded in the *Pendleton feedback style* supported by the learner centered models of feedback[12]. Constructivism theory demonstrates this by explaining how a learner constructs knowledge basing on their experiences through critical reflection on their performance[14].Moreover, active student reflection and constructive feedback from clinical instructors is a pillar of effective workplace learning which is critical to the development of student competence and confidence[6].

According to the tutors’ reports, many students did not act upon the feedback they received, resulting in the need for repetitive delivery of the same feedback. Additionally, some students rejected negative feedback, perceiving it as criticism. This finding could be attributed to the nature of feedback the teachers provided since it was not directly observed nor gave room for student reflection. These two critical aspects of feedback can motivate students into acting on the feedback provided. However, some studies[12] also note that that students tend to reject or ignore constructive feedback on their respective deficiencies or areas of improvement if there’s perceived lack of credibility in the process or provider.

Some teachers acknowledged a gap in their feedback process attributable to the lack of a standardized approach to giving feedback. A similar finding in [12] where teachers reported lack of feedback skills. Furthermore, the diverse range of students with different learning styles presented a challenge in delivering personalized feedback.

Overall, most of the students expressed dissatisfaction with the absence of feedback from their teachers; that the tutors only seemed concerned with monitoring their attendance during rotations, without providing any feedback. For the few teachers who did provide feedback, it was often inadequate as it would be delayed and inconsistent. Moreover, the students reported that some of the feedback they received was discouraging, consisting mainly of negative comments without any positive encouragement. Additionally, some teachers used harsh language in front of the patients, which had a negative impact on the students’ confidence. Similar results were found in a study by[10] conducted at the University of Johannesburg in South Africa. Such feedback delivered inappropriately or perceived to threaten learner’s self, can have a debilitating effect on motivation and performance [8]

Based on the demographics of the teachers/tutors in this study, most of them were fresh graduates and had been teaching for only 3 years and below(new to teaching); it should be noted that doctors or health professionals don’t automatically become teachers by virtue of their expertise or training, but a reflective approach to teaching and professional developments can foster excellence in workplace learning /clinical teaching[7].Implementing appropriate strategies like faculty development programs, institutions can enhance the impact of feedback on student learning and growth in clinical settings. Moreover, a competency-based curriculum like CMT,requires ongoing performance-based feedback for professional growth.

The strength of this study lay in the collection of data during ongoing clinical rotations, which helped to eliminate recall bias. Also collecting data from both the feedback provider (tutors) and recipient(students).But could have been limited by the fact that data was collected through tutors’ reported speech, which may not fully reflect the actual practices, a call for further research. More over the study was limited to only one institution, KIUT, potentially restricting the generalizability of the findings to other institutions.

Therefore, KIUT as an institution ought to provide the necessary orientation and training for their tutors on effective feedback practices. Although there are no internationally known standards/guidelines for feedback practices in a clinical rotation, there are several models to refer to for guidance. For example, Pendleton feedback model, the learner centered models of feedback that places the learner at the center of the feedback process[15]

## Conclusion

The study concluded that the feedback process under evaluation was incomplete as it lacked the two critical components of feedback that is; observing students’ performance and students’ reflection on their performance; hence the significant student dissatisfaction and highlighted challenge that was reported by almost all the tutors that students were not acting on the feedback provided. The lack of direct observation may have implications for accurately assessing students’ abilities and providing targeted feedback.

## Figures and Tables

**Table 1:** Demographic characteristics of the participants

TUTORS’ DEMOGRAPHICS		STUDENTS’ DEMOGRAPHICS	
Age (years)		Age (years)	
25–30	10	21	01
30–35	03	22	04
>35	01	23	07
Experience in teaching (years)		24	04
0–3	11	>24	01
4–6	02	MALES	12
>6	01	FEMALES	10

**Table 2:** The themes emerged from the study as tabulated below with their corresponding codes;

Themes	Codes	
	Tutors’ feedback practices	Students’ experiences of the feedback provided
Theme 1-Feedback delivery and approach	Instructive, observation-based assessment, collaborative, constructive, recognition and encouragement.	Valuable and focused. Good feedback and availability of some teachers. Beneficial feedback and teacher’s role. Regular feedback and guidance on improvement.
Theme 2-Feedback timing	Immediate after case presentations, during student-patient interactions and formal assessments.	Delayed and limited feedback time.Incosistent feedback.
Theme 3-Feedback content	addressing specific areas of improvement, assessing understanding, addressing questions, and tailoring feedback accordingly, assessment of clinical skills	Discussion and case-based feedback. Variety of feedback and different approaches. Harsh feedback in open and uncomfortable settings. Mixed experiences with feedback from hospital doctors. Unclear feedback and negative criticism.
Theme 4-Engagement and participation	Student involvement in the feedback process, promoting collaboration and active participation. Feedback in group setting, collaborative learning	Limited interaction and opportunities. Group dynamics and patient distribution.
Theme 5-Challenges	Students not being receptive to feedback. Not diligently completing assignments. Individual differences. Diverse learning styles. Student resistance to act on feedback. Repetition of discussions. Time constraints. Lack of active student participation. Absenteeism. No feedback guidelines. Group dynamics. Student response to criticism.	Lack of cooperation and support from clinicians. Realism and discrepancies in practice. Limited opportunities due to multiple colleges at clinical sites. Absent teachers during critical moments. Mixed good and bad feedback. Discouraging and negative feedback.

## Data Availability

The datasets generated and analyzed during this study are available in the Makerere University repository, http://hdl.handle.net/10570/12417.

## Abbreviations

KIUT: Kampala International University in Tanzania
CMT: Technician Certificate in Diploma
SN: serial number of a participant. (SNt=tutor. SNs=student)
